# Hydroxychloroquine therapy and its impact on bone mineral density among systemic lupus erythematosus patients: A cross-sectional study

**DOI:** 10.1515/rir-2026-0017

**Published:** 2026-08-02

**Authors:** Awalia, Stefanus Gunawan Kandinata, Arinditia Triasti Putri, Abellia, Lilik Maulidyatus Sholikhah, Rosy Setiawati, Mandana Nikpour

**Affiliations:** Division of Rheumatology, Department of Internal Medicine, Dr. Soetomo General Academic Hospital, Surabaya, Indonesia; Division of Rheumatology, Department of Internal Medicine, Faculty of Medicine – Universitas Airlangga, Surabaya, Indonesia; Department of Internal Medicine, Faculty of Medicine – Widya Mandala Catholic University, Surabaya, Indonesia; Department of Radiology, Faculty of Medicine – Universitas Airlangga, Surabaya, Indonesia; Department of Rheumatology, St Vincent’s Hospital, Fitzroy, Australia

**Keywords:** systemic lupus erythematosus, hydroxychloroquine, human & medicine, bone mineral density, osteoporosis

## Abstract

**Background and Objectives:**

Patients with systemic lupus erythematosus (SLE) are at increased risk of osteoporosis due to chronic inflammation, prolonged glucocorticoid (GC) exposure, and treatment-related factors. Hydroxychloroquine (HCQ), a cornerstone of SLE therapy, has shown conflicting effects on bone health. This study examined the association between HCQ use and bone mineral density (BMD) in SLE.

**Methods:**

A cross-sectional study of 213 SLE patients was conducted at Dr. Soetomo General Academic Hospital. BMD was assessed by dual-energy X-ray absorptiometry (DXA) and classified using WHO criteria. Demographic, clinical, laboratory, and treatment-related variables were collected. Group comparisons employed *chi-square* and non-parametric tests, while ordinal logistic regression identified independent predictors of reduced BMD.

**Results:**

Among 213 patients, 22.5% had normal BMD, 51.2% osteopenia, and 26.3% osteoporosis. Independent predictors of reduced BMD were older age (OR 1.05, 95% CI 1.01–1.09), higher cumulative GC exposure (OR 1.07, 95% CI 1.02–1.12), and lower BMI (OR 0.89, 95% CI 0.80–0.98). Importantly, the use of HCQ has been linked to a protective effect against osteoporosis (OR 0.47, 95% CI 0.22–0.97, *P* = 0.040). Neither SLEDAI scores nor complement/ESR levels were significantly associated with BMD.

**Conclusion:**

Low BMD is highly prevalent in SLE and is strongly influenced by age, GC exposure, and BMI. HCQ use was linked to a protective effect on bone health, plausibly mediated by anti-resorptive, antioxidant, and glucocorticoid-sparing properties. Although a causal relationship between these two variables cannot yet be established, this association underscores the need for further well-designed longitudinal or interventional studies.

## Introduction

Systemic lupus erythematosus (SLE) is a chronic, multisystem autoimmune disease marked by relapsing-remitting inflammation that leads to progressive tissue and organ damage. Recent studies indicate that patients with SLE carry an approximately 2.5-fold increased risk of osteoporosis compared to age- and sex-matched controls.^[1]^ As such, SLE management commonly involves combination therapies—most notably glucocorticoids (GCs), hydroxychloroquine (HCQ), and various immunosuppressants.^[2,3]^

Chronic systemic inflammation and prolonged GC use are well-established drivers of accelerated bone loss and increased fracture risk in SLE.^[1,4]^ There is also concern that other immunosuppressive agents may exacerbate bone fragility, particularly with long-term use.^[5,6]^ As a cornerstone of SLE therapy, HCQ exerts anti-inflammatory, antithrombotic, and cardioprotective effects.^[2,3]^ However, its specific impact on bone mineral density (BMD) remains unclear. *In vitro* and animal studies have shown heterogeneous results: some report that HCQ and chloroquine (CQ) reduce bone resorption by mechanisms such as receptor activator of nuclear factor-κB ligand (RANKL) inhibition, upregulation of Nrf2, and reduction of reactive oxygen species,^[7,9]^ while others document impaired osteoblast function or reduced BMD in rodent models.^[9,10]^

Human data are also inconsistent. One review cited moderate evidence that HCQ may improve bone mass in SLE and related conditions.^[11]^ Other observational studies in rheumatoid arthritis (RA) patients found that HCQ, either alone or combined with immunomodulators, increased spinal and hip BMD over one year.^[12,13]^ In contrast, a pediatric-onset SLE study revealed a negative association between HCQ cumulative dose and lumbar spine BMD,^[10]^ and a recent RA analysis found no association between HCQ use and osteoporosis risk.^[12,13]^ Given these discrepancies, the objective of the present study was to examine the relationship between HCQ therapy and bone mineral density in SLE patients, compared to those not using HCQ.

## Methods

### Study Design

This study employed a cross-sectional analytical design to investigate the association between HCQ therapy and BMD in patients with SLE. Data for the analysis were obtained from the rheumatology clinic database and patient medical records.

### Population and Sample

The study population consisted of patients diagnosed with SLE according to the American College of Rheumatology (ACR) 2019 classification criteria. Patients with comorbidities that could independently affect bone metabolism, such as advanced chronic kidney disease, thyroid disorders, or malignancy, were excluded. Total sampling method was used. In total, 213 patients were analyzed. Bone mineral density was measured using dual-energy X-ray absorptiometry (DXA) and was categorized according to World Health Organization (WHO) criteria into three groups: normal BMD (T-score ≥ –1.0), osteopenia (T-score between –1.0 and –2.5), and osteoporosis (T-score ≤ –2.5).

### Evaluated Parameters

The primary outcome variable was BMD status. Independent variables included demographic characteristics such as age, sex, and body mass index (BMI); disease-related parameters including disease duration and disease activity assessed by the Systemic Lupus Erythematosus Disease Activity Index (SLEDAI) at baseline and at the time of BMD assessment; laboratory markers including the lowest recorded complement C3 and C4 levels and erythrocyte sedimentation rate (ESR); and treatment-related factors, namely cumulative methylprednisolone dose, use of HCQ therapy and other immunosuppressant therapy.

### Statistical Analysis

All statistical analyses were performed using IBM SPSS Statistics (version 26). Continuous variables were summarized as mean ± standard deviation (SD) or median with interquartile range (IQR). Categorical variables were presented as absolute frequencies and percentages. Comparisons between the three BMD categories (normal, osteopenia, osteoporosis) were made using the Kruskal-Wallis test followed by post-hoc Dunn’s method with Bonferroni correction for pairwise comparisons where appropriate, and the Chi-square test for categorical variables. To identify independent predictors of reduced BMD, ordinal logistic regression was performed. Variables with a *p*-value < 0.25 and those considered clinically relevant were entered into the regression model. A p-value of less than 0.05 was considered statistically significant.

## Result

### Baseline Characteristics

A total of 213 patients with SLE were included, comprising 48 patients (22.5%) with normal BMD, 109 patients (51.2%) with osteopenia, and 56 patients (26.3%) with osteoporosis (Table 1). The majority of participants were female (97.7%), with only five male patients across all groups.

**Table 1:** Baseline characteristics of study sample

Baseline Characteristics	Normal (*n* = 48)	Osteopenia (*n* = 109)	Osteoporosis (*n* = 56)	*P*-value
Age (years)	29.50 [19.00]^ab^	34.00 [17.00]^a^	39.50 [25.00]^b^	0.007
Gender, F/M				0.607
Female (%)	48 (100%)	106 (97.25%)	54 (96.43%)	
Male (%)	0	3 (2.75%)	2 (3.57%)	
BMI (kg/m^2^)	22.42 ± 3.56	22.10 [5.20]	21.25 ± 3.35	0.106
Disease duration (year)	3.00 [5.00]^c^	5.00 [6.00]^c^	4.00 [6.00]	0.037
Lupus Nephritis (%)	20 (41.67%)	37 (33.94%)	17 (30.36%)	0.464
Cumulative Dosage of Methylprednisolone (gram)	5.60 [8.30]; *n* = 35	7.20 [10.20]; *n* = 76	8.64 [10.90]; *n* = 41	0.124
Lowest C3 (mg/dL)	76.70 ± 35.07	82.19 ± 39.43	75.10 ± 36.10	
Lowest C4 (mg/dL)	17.00 [22.00]	20.00 [22.00]	20.00 [21.00]	0.917
ESR mm/h	37.00 [39.00]	31.50 [24.10]	41.73 ± 22.65	0.542
HCQ Therapy	37 (77.08%)	67 (61.47%)	31 (55.36%)	0.061
Cumulative HCQ dose (gram)	144.00 [376.56]	144.00 [360.00]	72.00 [288.00]	0.337
Immunosuppressant Therapy				
Cyclophosphamide	7 (14.58%)	6 (5.50%)	6 (10.71%)	0.150
Cyclosporine	6 (12.50%)	30 (27.52%)	17 (30.36%)	0.072
Mycophenolic acid	24 (50.00%)	38 (34.86%)	26 (46.43%)	0.137
Azathioprine	12 (25.00%)	30 (27.52%)	8 (14.29%)	0.157
Methotrexate	7 (14.58%)	17 (15.60%)	4 (7.14%)	0.303
SLEDAI				
Baseline	8.00 [5.00]	8.00 [4.00]	8.00 [2.00]	0.282
Post-therapy	4.00 [6.00]^d^	0.00 [4.00]	0.00 [4.00]^d^	0.011

Superscripts with the same letters indicate statistically significant differences between the compared groups based on post hoc Mann–Whitney tests with Bonferroni correction. BMI, body mass index; HCQ, hydroxychloroquine; SLEDAI, systemic lupus erythematosus disease activity index.

The median age increased across the BMD categories, from 29.5 years [IQR 19.0] in the normal BMD group, to 34.0 years [IQR 17.0] in the osteopenia group, and 39.5 years [IQR 25.0] in the osteoporosis group. BMI was comparable between groups, with mean values of 22.4 ± 3.6 kg/m^2^ in normal BMD, a median of 22.1 [IQR 5.2] in osteopenia, and 21.3 ± 3.4 kg/m^2^ in osteoporosis.

Disease duration also tended to be longer in patients with reduced BMD: the normal BMD group had a median of 3.0 years [IQR 5.0], compared to 5.0 years [IQR 6.0] in osteopenia and 4.0 years [IQR 6.0] in osteoporosis. Cumulative methylprednisolone dosage was higher in osteopenia and osteoporotic groups (median 7.2 g [IQR 10.2] and 8.6 g [IQR 10.9], respectively) compared with the normal BMD group (median 5.6 g [IQR 8.3]). Interestingly, lupus nephritis occurred more frequently in the normal BMD group (20 [41.67%]) than in osteopenia (37 [33.94%]) and osteoporosis group (17 [30.36%]).

Laboratory markers showed subtle differences. The lowest recorded C3 levels were similar across groups (76.7 ± 35.1 mg/dL in normal BMD, 82.2 ± 39.4 mg/dL in osteopenia, and 75.1 ± 36.1 mg/dL in osteoporosis). C4 concentrations, however, were reported as medians: 17.0 mg/dL [IQR 22.0] in normal BMD, 20.0 mg/dL [IQR 22.0] in osteopenia, and 20.0 mg/dL [IQR 21.0] in osteoporosis. The ESR was also elevated across all groups, with a median of 37.0 mm/h [IQR 39.0] in normal BMD, 31.5 mm/h [IQR 24.1] in osteopenia, and a mean of 41.7 ± 22.7 mm/h in osteoporosis.

Disease activity scores showed a pattern of partial improvement with therapy. Baseline SLEDAI values were similar across groups, with medians of 8.0 [IQR 5.0], 8.0 [IQR 4.0], and 8.0 [IQR 2.0] in normal, osteopenia, and osteoporosis, respectively. Current SLEDAI was lower overall, though still numerically higher in the osteoporosis group (median 0.0 [IQR 4.0]) compared with osteopenia (median 0.0 [IQR 4.0]) and normal BMD (median 4.0 [IQR 6.0]). In addition, HCQ therapy was commonly used, but its distribution varied across groups; with 37 patients (77.1%) in the normal group, 67 patients (61.5%) in osteopenia, and 31 patients (55.4%) in osteoporosis. A more detailed analysis of cumulative HCQ exposure revealed that patients with normal BMD and osteopenia exhibited greater cumulative doses (144.0 [376.6] and 144.0 [366.0] gram, respectively) than those with osteoporosis (72.0 [288.0] gram). Meanwhile, the use of other immunosuppressive agents showed considerable variation across BMD groups.

### Univariate Associations Between Variables and BMD Status

Chi-square tests were used for categorical variables. Sex did not differ significantly between groups (*P* = 0.607), with females comprising the majority in all categories. Both HCQ therapy and its cumulative dose were also not associated with BMD status (*P* = 0.061; *P* = 0.337, respectively). Neither lupus nephritis nor the usage of other imunosuppressants was significantly different across all categories.

There was a significant difference in age across three BMD groups, χ^2^ (2, *n* = 213) = 9.87, *P* = 0.007. The median age was 29.50 for normal groups, 34.00 for osteopenia groups, and 39.50 for osteoporosis groups. Post-comparisons using Dunn’s method with a Bonferroni correction indicated that the median age of subjects in the osteopenia groups, *P* = 0.031 and osteoporosis groups, *P* = 0.008. However, there was no significant difference of age between osteopenia and osteoporosis groups, *P* = 1.000.

Disease duration was also significantly different across three BMD groups, χ^2^ (2, *n* = 213) = 6.60, *P* = 0.037. Post-hoc analysis showed that subjects in the osteopenia groups had significantly longer disease duration than the normal groups (*P* = 0.049). Meanwhile, no significant difference was found in the other subgroups.

Baseline SLEDAI scores were not significantly different (*P* = 0.282), whereas current SLEDAI scores were significantly different across BMD groups, χ^2^ (2, *n* = 209) = 9.06, *P* = 0.011. Post-hoc analysis showed a significant difference only between the normal and osteoporosis groups, with the osteoporosis group having lower SLEDAI scores (0.00 *vs*. 4.00).

Complement levels and inflammatory markers showed no significant associations with BMD. The lowest recorded C3 levels did not differ across groups (*P* > 0.05), C4 concentrations were similar (*P* = 0.917), and ESR was also comparable (*P* = 0.542). Cumulative glucocorticoid dose and BMI also showed no significant difference across BMD (*P* = 0.124 and 0.106, respectively).

### Independent Predictors of Reduced BMD in Multivariable Analysis

Ordinal logistic regression was performed, including variables significant in univariate tests (*P* < 0.25) and clinically relevant factors (Figure 1). Older age was an independent predictor of reduced BMD (OR = 1.05, 95% CI: 1.01–1.09, *P* = 0.007), as was higher cumulative methylprednisolone dose (OR = 1.07, 95% CI: 1.02–1.12, *P* = 0.008). Higher BMI was protective (OR = 0.89, 95% CI: 0.80–0.98, *P* = 0.024).

Other parameters, including disease duration (OR = 0.89, 95% CI: 0.78–1.01, *P* = 0.080), current SLEDAI, lupus nephritis, and the use of immunosuppressants (cyclophosphamide, cyclosporine, MPA, and azathioprine) were not independently associated with BMD. Notably, HCQ therapy was significantly protective towards incidence of osteoporosis (OR = 0.47, 95% CI: 0.22–0.97, *P* = 0.040).

**Figure 1:**
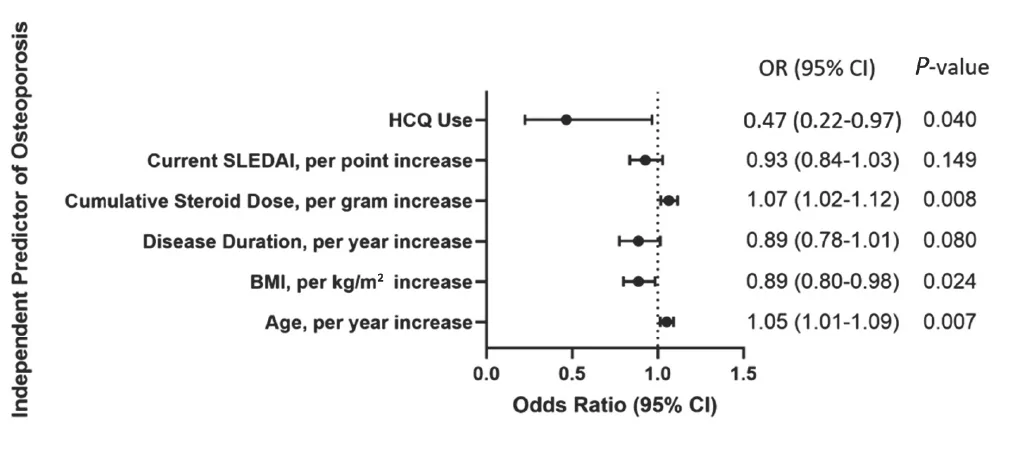
Independent predictors of osteoporosis.

## Discussion

In this cohort of 213 patients with SLE, more than three-quarters had either osteopenia or osteoporosis, reaffirming the high prevalence of low BMD in this population.^[1,3,14]^ Older age, cumulative glucocorticoid (GC) exposure, and lower BMI emerged as the strongest predictors of reduced BMD, consistent with established mechanisms including impaired osteoblast function, postmenopausal hormonal decline, and GC-mediated suppression of bone formation.^[4, 5, 6]^

The most important finding of this study was the protective association of HCQ with bone health. Although a causal relationship between these two variables cannot yet be established, patients not receiving HCQ had more than twice the odds of reduced BMD compared with those on therapy, suggesting that HCQ associate with skeletal preservation. This observation is biologically plausible, as HCQ has been shown to inhibit osteoclastogenesis and bone resorption *in vitro* and *in vivo*,^[7]^ and to prevent bone loss in preclinical models.^[8]^ Furthermore, clinical data in female SLE patients demonstrated that HCQ use was independently associated with higher hip and spine BMD,^[4]^ while reviews have also highlighted its potential bone-protective role within autoimmune disease management.^[12]^

Mechanistically, HCQ may protect bone through multiple pathways. HCQ has been shown to reduce osteoclast differentiation and activity by interfering with the RANKL signaling pathway.^[7]^ Inhibition of RANKL reduces osteoclast formation and bone resorption, a central mechanism in the pathogenesis of osteoporosis. HCQ may also upregulate nuclear factor erythroid 2–related factor 2 (Nrf2), enhancing antioxidant defenses and decreasing reactive oxygen species (ROS) –mediated bone loss.^[7,12]^ By dampening oxidative stress, HCQ can prevent apoptosis of osteoblasts and osteocytes, thereby maintaining bone formation capacity. Experimental models further suggest that HCQ modulates autophagy and lysosomal activity, which may regulate bone cell survival and turnover.^[8,9]^

Another important mechanism is the indirect skeletal benefit mediated through HCQ’s systemic immunomodulatory actions. HCQ suppresses toll-like receptor activation and reduces production of proinflammatory cytokines such as TNF-α and IL-6, both of which stimulate osteoclastogenesis and bone resorption.^[6,12]^ By lowering systemic inflammation, HCQ contributes to a less catabolic bone environment. In addition, HCQ is known to have a glucocorticoid-sparing effect in SLE, thereby reducing cumulative GC exposure—one of the strongest predictors of bone loss in autoimmune diseases.^[5,6]^ Through these combined pathways—direct inhibition of bone resorption, protection of bone-forming cells, antioxidant effects, and indirect GC-sparing properties—HCQ plausibly contributes to improved skeletal outcomes in SLE. Nevertheless, evidence from other contexts remains mixed. A large rheumatoid arthritis cohort found no significant reduction in osteoporosis risk with HCQ,^[11]^ indicating that benefits may be disease-specific or influenced by confounding factors. However, these findings do not negate the potential protective effect in SLE, especially when considering the established anti-inflammatory effect of HCQ.

In our study, disease activity scores (SLEDAI) and inflammatory markers (C3, C4, ESR) were not predictive of BMD, consistent with findings from the Kyoto Lupus Cohort and the Toronto Lupus Cohort.^[13,14]^ This suggests that long-term exposures—particularly GC burden and HCQ therapy—are stronger determinants of bone health than short-term disease activity measures. Although neither lupus nephritis (LN) nor immunosuppressant use was independently associated with BMD in our multivariable analysis, both may plausibly influence skeletal health through indirect and heterogeneous mechanisms. LN may adversely affect bone *via* a renal–bone axis, including greater cumulative glucocorticoid exposure, persistent systemic inflammation, impaired kidney function with features of chronic kidney disease–mineral and bone disorder, proteinuria-associated vitamin D losses, secondary hyperparathyroidism, and reduced physical activity.^[15,16]^ The absence of a significant association in our study may be partly attributable to the exclusion of patients with advanced chronic kidney disease and to the binary classification of LN, as prior studies have demonstrated stronger associations when LN is stratified by histological class, disease activity or chronicity indices, remission status, proteinuria burden, or estimated glomerular filtration rate. In addition, contemporary guideline-recommended steroid-sparing treatment strategies for LN may indirectly mitigate bone loss, although renal and mineral metabolism biomarkers were unavailable in the present study.^[15]^

Similarly, immunosuppressant exposure is inherently heterogeneous, with individual agents exerting distinct and often indirect skeletal effects that are frequently confounded by underlying disease severity and glucocorticoid burden. For example, cyclophosphamide may contribute to bone loss through premature ovarian failure and early menopause,^[17]^ while calcineurin inhibitors have been associated with increased bone turnover and potential bone loss in some settings, although their clinical impact on BMD appears to be strongly dependent on concomitant steroid exposure and disease control.^[18,19]^ Furthermore, the lack of an association between cumulative HCQ dose and BMD, despite the observed protective effect of HCQ use, may reflect limitations of cumulative exposure as a surrogate for biologically relevant dosing. A large population-based study reported no significant association between HCQ treatment duration and osteoporosis risk, even at higher cumulative exposure levels.^[20]^ The effects of HCQ on bone metabolism are complex and heterogeneous, with variable effects on bone formation and resorption depending on dose, duration, and biological context.^[21]^ Additionally, longer disease duration is a recognized risk factor for reduced bone mass and fractures in systemic autoimmune diseases, complicating simple associations between cumulative drug exposure and BMD.^[3,4]^ Early observational and mechanistic data suggesting a protective association between HCQ use and bone health further underscore that commonly used HCQ exposure metrics may not capture clinically relevant effects on skeletal outcomes.^[7,12]^

Clinically, these results reinforce the importance of comprehensive bone health strategies in SLE. DXA screening should be prioritized for patients with traditional risk factors such as older age, low BMI, and chronic GC exposure. Given its favorable safety profile and its association with preserved BMD in our analysis, HCQ should remain a cornerstone therapy in SLE, offering not only disease control but also a potential skeletal benefit. While prior findings across autoimmune diseases remain heterogeneous, the balance of evidence justifies continued HCQ use in SLE, alongside preventive measures such as lifestyle optimization and judicious GC use.^[14]^

This study has several strengths, including its relatively large sample size, the use of standardized DXA assessments, and the application of multivariable modeling to minimize confounding. However, some limitations should be acknowledged, including the cross-sectional design, which precludes causal inference, the absence of longitudinal follow-up, and the lack of bone turnover markers or fracture outcomes. Taken together, while HCQ appears promising for skeletal protection in SLE, these strengths must be interpreted in light of study limitations and the need for prospective validation. These limitations also highlight important opportunities, such as future prospective studies to validate the protective role of HCQ, clarify its underlying mechanisms, and determine whether it reduces fracture incidence in SLE. At the same time, potential threats must be considered, including heterogeneity in prior clinical findings and the risk of residual confounding by disease severity or treatment history.

## Conclusion

Low BMD is highly prevalent in systemic lupus erythematosus and is strongly influenced by age, glucocorticoid exposure, and BMI. Our findings demonstrate that HCQ use is associated with a protective effect on BMD, plausibly mediated by its anti-resorptive, antioxidant, anti-inflammatory, and glucocorticoid-sparing properties. Given its favourable safety profile and multiple systemic benefits, hydroxychloroquine should remain a cornerstone of SLE therapy, with the potential associations to osteoporosis prevention, while future prospective studies are needed to confirm its causal role and impact on fracture outcomes.
